# Adaptation of *Glycyrrhiza glabra* L. to water deficiency based on carbohydrate and fatty acid quantity and quality

**DOI:** 10.1038/s41598-023-28807-6

**Published:** 2023-01-31

**Authors:** Tahereh Movahhed Haghighi, Mohammad Jamal Saharkhiz, Gholamreza Kavoosi, Mehdi Zarei

**Affiliations:** 1 Department of Horticultural Science, Faculty of Agriculture, Shiraz University, Shiraz, 71441-13131 Iran; 2 Medicinal Plants Processing Research Center, Shiraz University of Medical Sciences, Shiraz, Iran; 3 Institute of Biotechnology, Shiraz University, Shiraz, 71441-65186 Iran; 4 Department of Soil Science, College of Agriculture, University of Shiraz, Shiraz, Iran; 5 Department of Agriculture and Natural Resources, Higher Education Center of Eghlid, Eghlid, 73819-43885 Iran

**Keywords:** Biochemistry, Plant sciences, Biogeochemistry

## Abstract

Water deficit affects agricultural systems negatively globally. This research objective was to mitigate drought’s detrimental effects on plants metabolite profiling by utilizing biofertilizers and mineral nutrition. The carbohydrate content and fatty acid profile of Licorice (*Glycyrrhiza glabra*) were assessed under Silicon (Si) nutrition, *Claroiedoglomus etunicatum* inoculation (F), and drought stress (100, 80, 60, 40, and 20% of field capacity (FC)). Results showed that Si application increased total sugar content under severe drought levels (20 and 40% FC) and made it reach 12.41 and 12.63 g/100 g _DW_, respectively. Sucrose, as the predominant sugar of licorice, was at its highest level (13.1 g/100 g _DW_) in response to integrated values of F and Si (60% FC). Gas chromatography–mass spectrometry showed that the majority of fatty acid components in plants were 9-Octadecenoic acid (8.72–71.27%), 9,12-Octadecadienoic acid (0.1–56.43%), Hexadecanoic acid (12.84–30.59%), Octadecanoic acid (6.9–15.3%), Docosanoic acid (0.57–2.77%), Eicosanoic acid (1.07–2.64%), and 7-Hexadecenoic acid (0.26–2.62%). Since a lower omega6/omega3 ratio represents a healthier product, the lowest ratio (0.25%) was observed in well-watered inoculated plants. Also, severe drought-treated plants under integrated Si and F applications showed a low omega6/omega3 ratio (1.88%). In conclusion, Si and F improved synergistically the carbohydrate content and fatty acid profile in plants, despite the drought stress.

## Introduction

Licorice (*Glycyrrhiza glabra* L. family: Fabaceae) is a valuable medicinal plant which is commonly processed in the biopharmaceutical and nutraceutical industries^1^. It is also considered as a food additive because of its high sweetness. Careless overexploitation of wild ecotypes of *G. glabra* has recently resulted in a rapid reduction of its populations, if not extinction. Because of the plant’s high economic value and the risk of extinction owing to overharvesting, it appears vital to grow and domesticate this valuable species^2^. It has various physiologically-active compounds, including terpenes, flavonoids, polysaccharides, sugars, amino acids, minerals, lipids, and glycosides^3^. Recently, polysaccharides have been identified with distinctive biological functions, including antimicrobial and immunomodulation effects^4^. Numerous studies have suggested that licorice polysaccharides can regulate immunity, while having antiviral and antioxidant activities, with minimal cytotoxic effects. Since this valuable medicinal-industrial plant has been harvested indiscriminately, it is endangered and needs to be cultivated. Since many cultivated areas are facing water deficits as a widespread abiotic stress, the deficit has negatively affected plant productivity and disrupted normal metabolism^5^. Drough has many detrimental effects on quantity and quality of plant production systems. As a result of prolonged drought, cell turgor maintenance happens via the buildup of organic osmolytes including carbohydrates, sugars, and fatty acids^6^. Sugars, as one of the main components of licorice roots, are osmotic adjustors and signaling molecules, capable of being produced by carbohydrate metabolism, as they activate a variety of protective reactions to help tolerate drought stress. Sugars are also known to provide essential carbon content and energy for development, cell proliferation, differentiation, and preservation^7^. Also, plant sucrose, which is high in licorice root extract, and its products that result from hydrolysis, as well as products such as fructose and glucose transporters, can regulate their redistribution under abiotic stress between source and sink^8^. So, when water-deficit conditions exist, sugar transportation occurs in the roots by upregulating the expression of some transporter genes. Furthermore, the composition of fatty acids and biosynthesis are often altered. These various harmful impacts of water deficit on basic metabolites of licorice, carbohydrates and fatty acids, affect their critical roles on licorice quality for food and pharmaceutical industries. Wild licorice plants are well-suited to growing in challenging situations, including drought, and on lands with nutrient deficiency^9^. These are the two main causes that usually limit licorice production. *Glycyrrhiza* plants are grown to help restore ecosystems that have been degraded, especially in arid and semi-arid areas. In this regard, plants have evolved a variety of stress-resistance strategies. Plant–microbe mutualism may influence plant development, nutrient uptake, and resistance to water-deficit stress. So, there are several ways to mitigate these negative impacts of drought stress on the quantity of carbohydrates and the profile of lipids^10^. Since some minerals can reduce the effects of stresses, including drought stress, Si was used in the present study. Mineral elements such as exogenous Si can partly neutralize the negative impact of water-deficit on plant metabolites^11^. Numerous mechanisms are described in improvements through Si-mediated growth, including the activation of photosynthetic enzymes and enzymatic antioxidant defense systems, an enhanced water use efficiency, better nutrient uptake, regulating stomatal behavior and hydraulic conductance, regulate aquaporins, and the accumulation of organic osmolytes^4^. Although Si is often not regarded as an essential element, previous research have shown that it helps mitigate abiotic stress in plant species such as *Lens culinaris*^12^, *G. glabra*^9^, and *Glycyrrhiza uralensis*^13^. Another strategy to reduce the effects of drought stress on the quantity and quality of plant metabolites is to use biofertilizers such as mycorrhiza. Arbuscular mycorrhizal fungi (AMF) are the broadest genus capable of symbiosis in the plant kingdom. They are another strategy to improve plant metabolomics under irrigation regimes^14^. AMF is considered as a key player in agronomic practices, as it has important components for sustainable management in agricultural ecosystems^15^. AMF-colonization can improve the establishment of extensive hyphal networks which assist in water absorption, and leading to better soil structures. AMF colonization of roots can improve plant development by enhancing nutrient uptake, ion homeostasis, root development, accumulating osmolytes, induction of drought-responsive genes, and activation of different metabolic pathways^16^. Previous cases of research on *Ceratonia silique*^17^ and *G. glabra*^9^ have already explained the positive effects of AMF on plant drought resistance and metabolite accumulation.

In the present study, a pot experiment was carried out to evaluate the impacts of AMF and Si, individually and in combination, on licorice carbohydrate content and on fatty acid profile, under different levels of drought stress.

## Materials and methods

### Plant materials

This study was carried out in the College of Agriculture, Shiraz University. The seeds of *G. glabra* (voucher number: MPH-2670-1) were collected from Eghlid area (Aspas village, 52° 23′ 58″ E and 30° 38′ 31″ N), a region in the north of Fars province, Iran. The collection was done following national and scientific guidelines as described by Esmaeili et al.^1^ and based on the International Standard for Sustainable Wild Collection of Medicinal and Aromatic Plants (ISSC-MAP) (Version 1.0) prepared by the Medicinal Plant Specialist Group of the IUCN Species Survival Commission (The World Conservation Union). Also the permission to collect seeds was obtained from the (Iranian Government Organization) Natural Resources and Watershed Management Organization. The seeds were scarified by soaking in concentrated H_2_SO_4_ (97%- Merck) for 10 min, washed with running water several times, and immediately sown in transplant trays with Peat Moss and perlite mix, 2:1^18^. Two seeds were sown in each cell of the trays. They were placed in the greenhouse (Day: 27 ± 1 °C, night: 23 ± 1 °C, humidity: 70 ± 3%, light: 40,000 Lux) after sowing. A month after germination, the seedlings were transplanted into small plastic pots containing 250 mL of field soil and sand mixture (2:1) (non-sterilized). Bigger pots were used for transplanting 6-month-old seedlings in a sandy medium (soil and sand mixture (2:1)).

### Mycorrhizal inoculation preparation

*Claroiedoglomus etunicatum* was provided by the soil biology lab at Shiraz University. It was previously separated by Dr. Mehdi Zarei in the Department of Soil Sciences. The inoculum (250 g) was added to the root zone as transplanting took place in the final pots. Each mycorrhizal pot received soil containing fungal spores, mycorrhizal roots, and mycelia of *C. etunicatum*, and the non-mycorrhizal pots received an equal amount of washed sand^19^.

### Growth situation and treatment application

After two weeks of adaptation and plant establishment in the pots, irrigation treatments were carried out for two months. Irrigation was performed to make the soil reach five different levels of moisture, either at or below field capacity (FC). These levels were either 20, 40, 60, 80, and 100% FC (control). Drought stress treatments are shown as “W”, as W_100_, W_80_, W_60_, W_40_, and W_20_. Silicon application is represented by “Si” at two levels, with Si (Si_1_) and without (Si_0_). Fungi inoculation was also shown by “F” at two levels, with (F_1_) and without (F_0_) fungi inoculation. Si was dissolved in the irrigation water as SiO_2_ at 300 mg/L concentration. As the plants consumed water and as evaporation occurred, the weight of pots decreased gradually through the course of observations^20^. After eight weeks, and under the respective conditions of growth, the plants were harvested for analysis.

### Total sugar extraction and determination

The quantification of total sugar in the roots and rhizomes of licorice was carried out according to a procedure in the available literature with little modifications^21^. A grinder was used for crushing the samples before extraction. In the extraction stage, 100 mg of dried-ground sample was poured into a microtube and then 2 mL ethanol (80%) was added. The solution was kept at room temperature a night. In being decanted into another 15-mL tube after centrifugation at 3000 rpm for 10 min, the residue was stored in the centrifuge tube. This extraction was repeated two more times. After that, 80% ethanol was added to the supernatant in a 15-mL volumetric flask. The total soluble sugar content of this extract was determined. Total soluble sugar was measured by adding 25 µL soluble sugar extract into a microplate cell. Then, the procedure was followed by the addition of 25 µL phenol (5%) and 125 µL sulfuric acid. The absorbance was read at 490 nm by an Epoch microplate spectrophotometer (USA)^21^.

### Starch extraction and determination

The residue from total sugar extraction, was used for starch determination according to a procedure in the available literature with little modifications^22^. The residue in each test tube, occurring from total sugar extraction, was dried at 80 °C for one h. Then, the tubes were filled with 200 µL cold distilled water and awaited complete absorption. Then, 260 µL of the tube was filled with perchloric acid (52%), and the tube was occasionally swirled with a vortex^15^. Again, 400 µL cold distilled water was added to the suspension and centrifuged at 3000 rpm (10 min). The supernatant was subsequently decanted into a 2 mL tube. This was followed by adding 100 µL cold distilled water and 130 µL perchloric acid (52%) to the residue of the test tubes. A vortex was used for swirling this suspension for 15 min. Centrifuged and decanted supernatants were mixed into the 2 mL tube. For starch analysis, 100 µL of starch extract were added into a microplate cell. Then, 200 µL of anthrone reagent was gradually added to the cells. The microplate was relocated into an oven (65 °C) where it remained for exactly 20 min. After cooling at ambient temperature, the absorbance was read at 630 nm by an Epoch microplate spectrophotometer (USA)^22^.

### Glucose, sucrose, and fructose profiling by HPLC

HPLC–RID was used for determining the free sugar contents individually. The isocratic Agilent 1100 HPLC method was used for identifying free sugars at 40 °C. The HPLC system was provided with an Agilent smart line RID detector and a carbohydrate column (4.6 × 250 mm, 5 mm, Agilent). At a flow rate of 1.5 mL/min, the mobile phase consisted of a deionized water/acetonitrile (20:80 v/v) composition. The injection volume amounted to 20 µL. An internal normalization of the chromatographic peak area was used for analyzing the data^23^.

### Preparation of oil extracts from licorice

The roots were harvested, cleaned from the soil, and dried at ambient temperature. Then, the dried roots were ground by an electric grinder. Licorice powder (500 mg) was suspended in a hydrolysis buffer (5.0 mL) and included normal saline: methanol: hydrochloric acid (1:1:2) which was mixed attentively and incubated at 70 °C for three days to allow the hydrolysis of the licorice biomass. In these conditions, proteins, lipids, and carbohydrates were digested to amino acids, fatty acids, and monosaccharides, respectively^24^. Accordingly, 3.0 mL hexane was added to normal saline: methanol: hydrochloric acid hydrolysate and vortexed for 10 min to allow the separation of fatty acids. The fatty acid that existed in the hydrolysate was separated from biomass overnight at ambient temperature. The fatty acid in the upper phase (hexane phase) was isolated and characterized chemically using GC–MS^25^.

### Fatty acids methyl esters (FAMEs) profiling by GC–MS

The GC–MS analysis was performed using an Agilent gas chromatography (Agilent 7890B GC 7955A MSD) equipped with a fused silica capillary HP-5MS column (30 m × 0.25 mm id; thickness 0.25 µm), coupled with a single quadrupole mass spectrometer. At a flow rate of 1.0 mL/min, helium was used as a carrier gas. The temperatures of the ion source and interface were 250 °C and 300 °C, respectively. The oven temperature program was set to increase from 80 to 240 °C as follows: 80 °C for 4 min, which rose to 140 °C at a rate of 20 °C/min. Thereafter, it reached 250 °C at 10 °C/min and was held at 240 °C for 10 min. By comparing the retention times and fragmentation patterns of the linked peaks with those described in the Wily 7n and NIST05a libraries, the GC–MS apparatus software rightly detected fatty acids^26^.

### Statistical analysis

The pot experiment was set up in a factorial arrangement with four replications in a completely randomized design. It comprised three factors including drought levels, AMF inoculation, and Si nutrition. The data were examined by the GLM test using Minitab software (Version 17; Available from: http://www.minitab.com/en-US/products/minitab/). In the case of significant interactions, the slice method was applied for mean comparisons. Tukey’s test at the 5% level operated to make mean comparisons. Then graphs created by Microsoft Excel software (Version 2016; Microsoft Corporation. Retrieved from https://www.microsoft.com/en-us/microsoft-365/excel). Minitab (Version 17; Available from: http://www.minitab.com/en-US/products/minitab/) was also employed to perform the principal component analysis (PCA).

## Results

### Total sugar quantity of licorice under mentioned treatments

**Figure 1:**
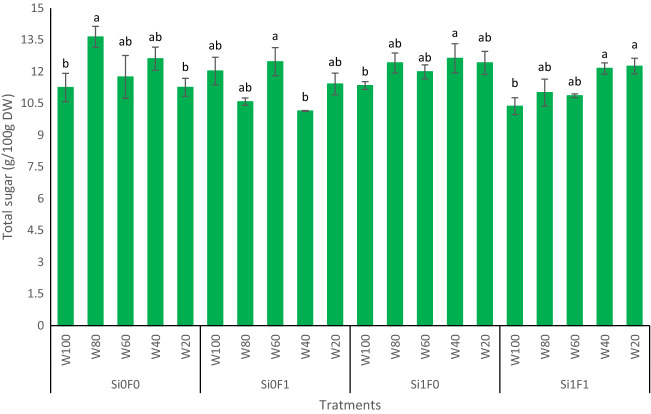
Total sugar content of licorice root under drought stress integrated by silicon nutrition and mycorrhiza inoculation. According to the analysis of variance that triple effects of fungi, drought levels and Si showed significant difference, slice method used for mean comparisons. Mean values with the same letters are not significantly different (*p* < 0.05), Tukey test. W stands for water deficit treatment (5 levels including 100% (W100), 80% (W80), 60% (W60), 40% (W40), and 20% (W20) of field capacity), Si stands for silicon application (two levels including Si0 (no Si application) and Si1 (Si application)) and F stands for mycorrhiza inoculation (two levels including F0 (no inoculation) and F1 (inoculated)).

In the present study, the total sugar content was significantly (*p*-value < 0.05) affected by the interaction among Si, F, and drought levels (Fig. 1). The results showed that exogenous Si assisted licorice plants significantly in maintaining soluble sugar content, despite severe drought stress levels (W_20_ and W_40_), compared to non-Si-treated plants that faced the same stress levels. In this regard, higher amounts of total soluble sugar were achieved (12.63 and 12.41 g/100 g _DW_) in response to W_40_Si_1_ and W_20_Si_1_, respectively. Furthermore, integrated Si and F inoculation ultimately increased the total sugar content (12.26 g/100 g _DW_) in the face of severe drought stress (W_20_), whereas F inoculation per se had a smaller effect (Fig. 1).

### Soluble carbohydrate profiling of licorice

**Figure 2:**
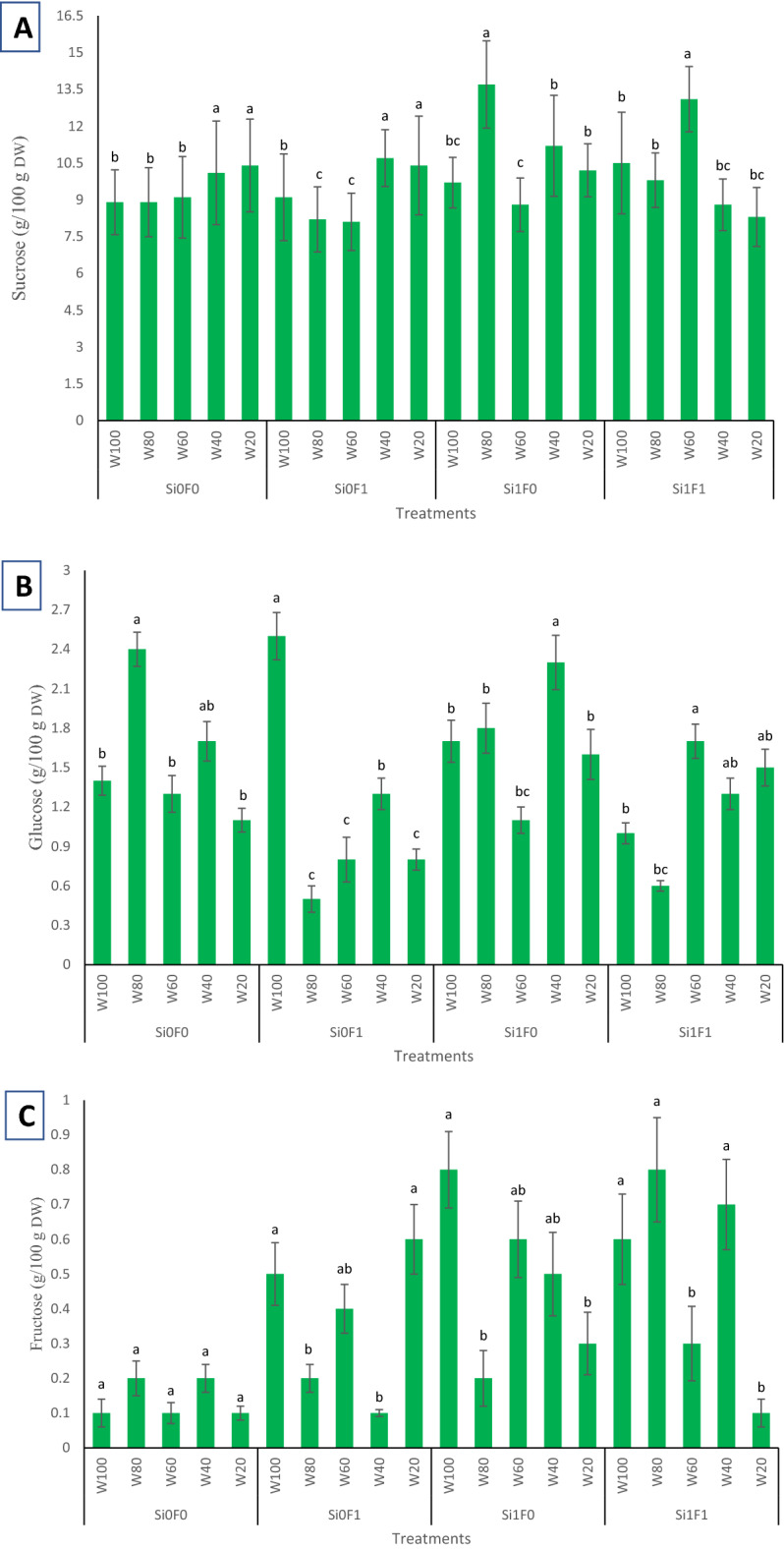
HPLC analysis of (**A**) sucrose, (**B**) glucose and (**C**) fructose among drought stress, Si application and mycorrhiza inoculation interactions in examined Licorice plants. According to the analysis of variance that triple effects of fungi, drought levels and Si showed significant difference, slice method used for mean comparisons. Mean values with the same letters are not significantly different in each treatment (*p* < 0.05), Tukey test. W stands for water deficit treatment (5 levels including 100% (W100), 80% (W80), 60% (W60), 40% (W40), and 20% (W20) of field capacity), Si stands for silicon application (two levels including Si0 (no Si application) and Si1 (Si application)) and F stands for mycorrhiza inoculation (two levels including F0 (no inoculation) and F1 (inoculated)).

HPLC profiling of soluble carbohydrates in licorice roots showed a high sucrose content (13.1 g/100 g _DW_) in response to the W_60_Si_1_F_1_ treatment, compared to all other inoculated plants and many other non-inoculated plants. High glucose contents were observed in plants of the W_100_Si_0_F_1_ (2.5 g/100 g _DW_) and W_80_Si_0_F_0_ (2.4 g/100 g _DW_). High contents of fructose in the present study were recorded in treatment groups of W_100_Si_1_F_0_ (0.8 g/100 g _DW_), W_80_Si_1_F_1_ (0.8 g/100 g _DW_), and W_40_Si_1_F_1_ (0.7 g/100 g _DW_). Irrespective of F inoculation, Si-treated licorice plants showed high sucrose contents of 11.2, 13.1, and 13.7 g/ 100 g _DW_ when situated in 40, 60, and 80% FC irrigation, respectively (Fig. 2A–C).

### Starch quantity of licorice under mentioned treatments

**Figure 3:**
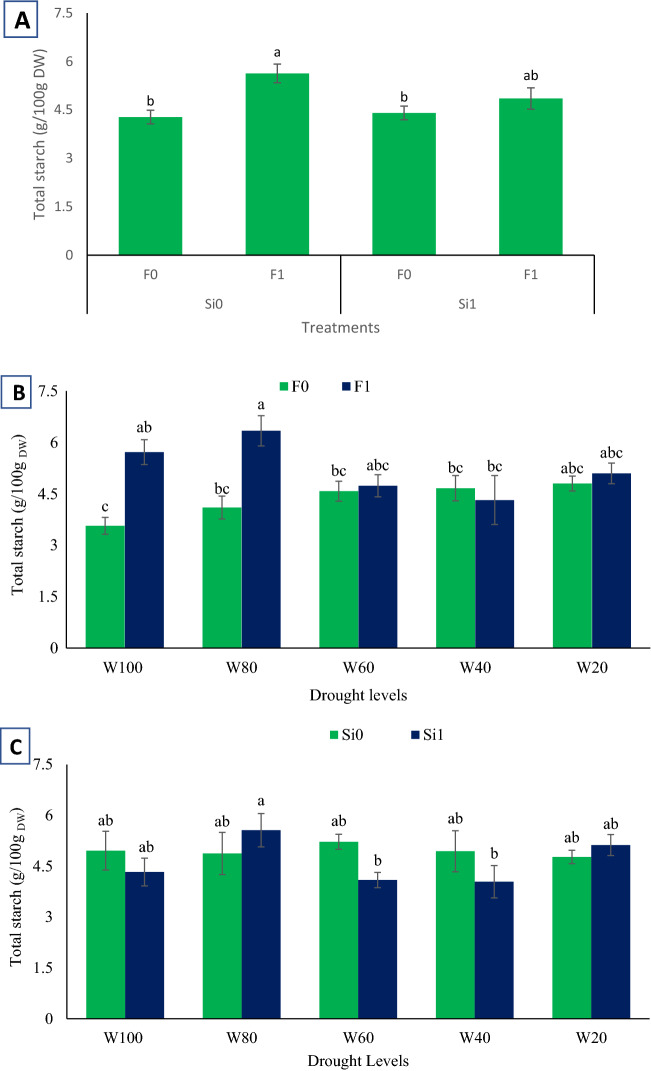
Total starch content variation among (**A**) Si application and fungi inoculation interactions, (**B**) various drought levels and fungi inoculation interactions and (**C**) various drought levels and Si application interactions in examined Licorice plants. According to the analysis of variance that only the multiple effects of fungi and Si showed significant difference, just its mean comparison is shown. Mean values with the same letters are not significantly different (*p* < 0.05), Tukey test. W stands for water deficit treatment (5 levels including 100% (W100), 80% (W80), 60% (W60), 40% (W40), and 20% (W20) of field capacity), Si stands for silicon application (two levels including Si0 (no Si application) and Si1 (Si application)) and F stands for mycorrhiza inoculation (two levels including F0 (no inoculation) and F1 (inoculated)).

In using the exogenous Si, the starch content in licorice roots varied among fungi-inoculated and non-inoculated plants. It was observed that inoculated treatments caused higher starch contents. Maximum starch content was observed in response to Si_0_F_1_ (5.63 g/100 g _DW_), while minimum quantities were observed in Si_0_F_0_ (4.27 g/100 g _DW_) and Si_1_F_0_ (4.4 g/100 g _DW_) (Fig. 3A).

In well-irrigated treatments (80 and 100% FC), there was a significant (*p*-value < 0.05) difference between the effects of F_0_ and F_1_, although such a difference was not caused by other drought levels. Through the interaction between W and F, a minimum starch value was observed in response to W_100_F_0_ (3.56 g/100 g _DW_), while a maximum was achieved in W_80_ F_1_ (6.33 g/100 g _DW_) (Fig. 3B). The results showed that the quantity of starch decreased significantly (*p*-value < 0.05) parallel to an increase in drought stress severity. Despite the effects of exogenous Si, the W_60_ and W_40_ treatments caused the lowest starch contents (4.09 and 4.04 g/100 g _DW_, respectively) (Fig. 3C).

### Fatty acid content and composition of licorice roots

**Figure 4:**
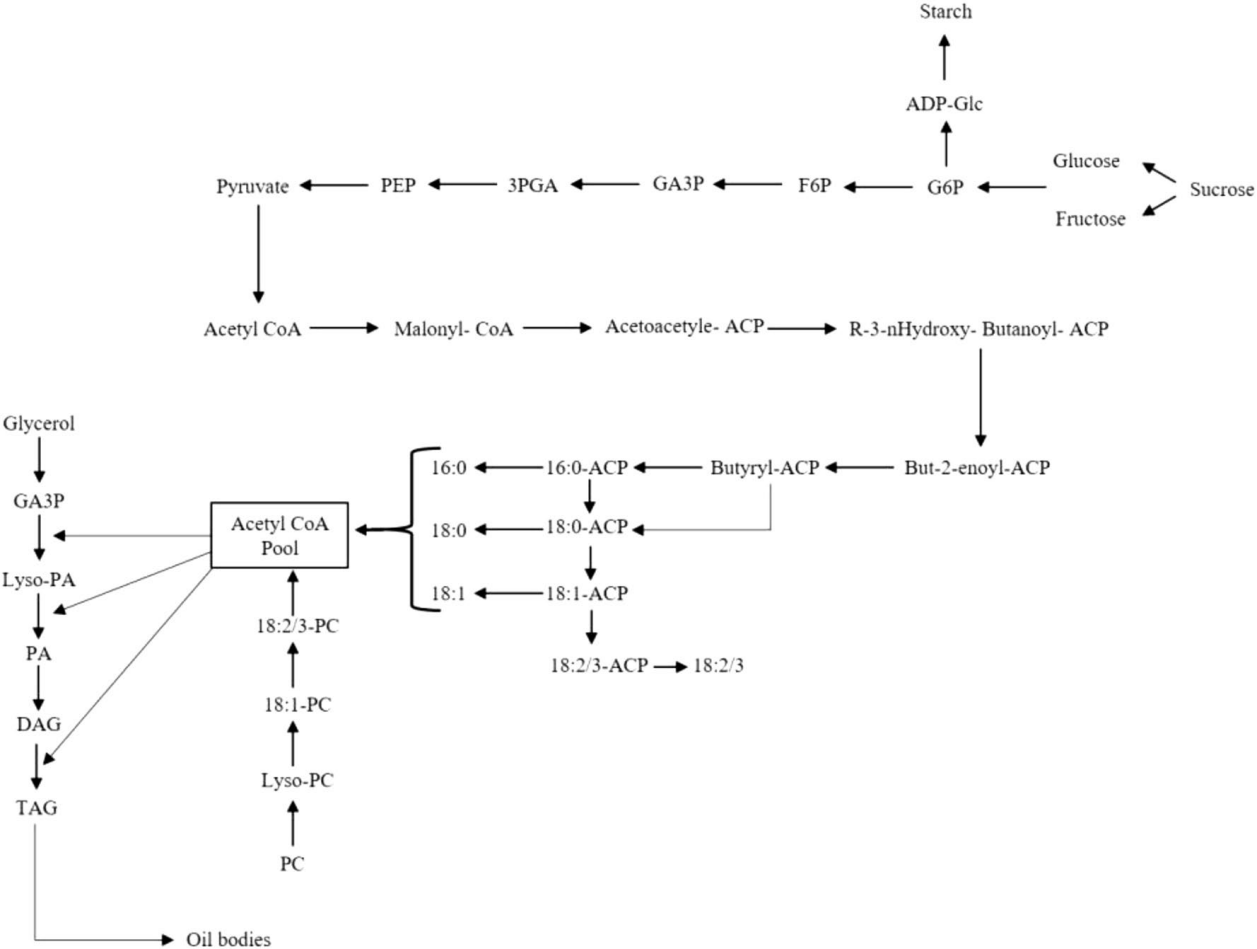
Integrated Carbohydrate and fatty acid biosynthesis pathways [Adapted from^59^ with modifications]. *G6P* glucose 6-phosphate, *ADP-Glc* ADP-glucose, *T6P* trehalose 6- phosphate, *F6P* fructose 6-phosphate, *GA3P* glyceraldehyde 3-phosphate, *3PGA* 3-phosphoglyceric acid, *PEP* phosphoenolpyruvate, *CoA* coenzyme A, *ACP* acyl-carrier protein, *Lyso-PC* lysophosphatidylcholine, *Lyso-PA* lysophosphatidic acid, *DAG* diacylglycerol, *TAG* triacylglycerol. This figure is created by Microsoft PowerPoint software (Version 2016; Microsoft Corporation. Retrieved from https://www.microsoft.com/en-us/microsoft-365/powerpoint).

**Figure 5:**
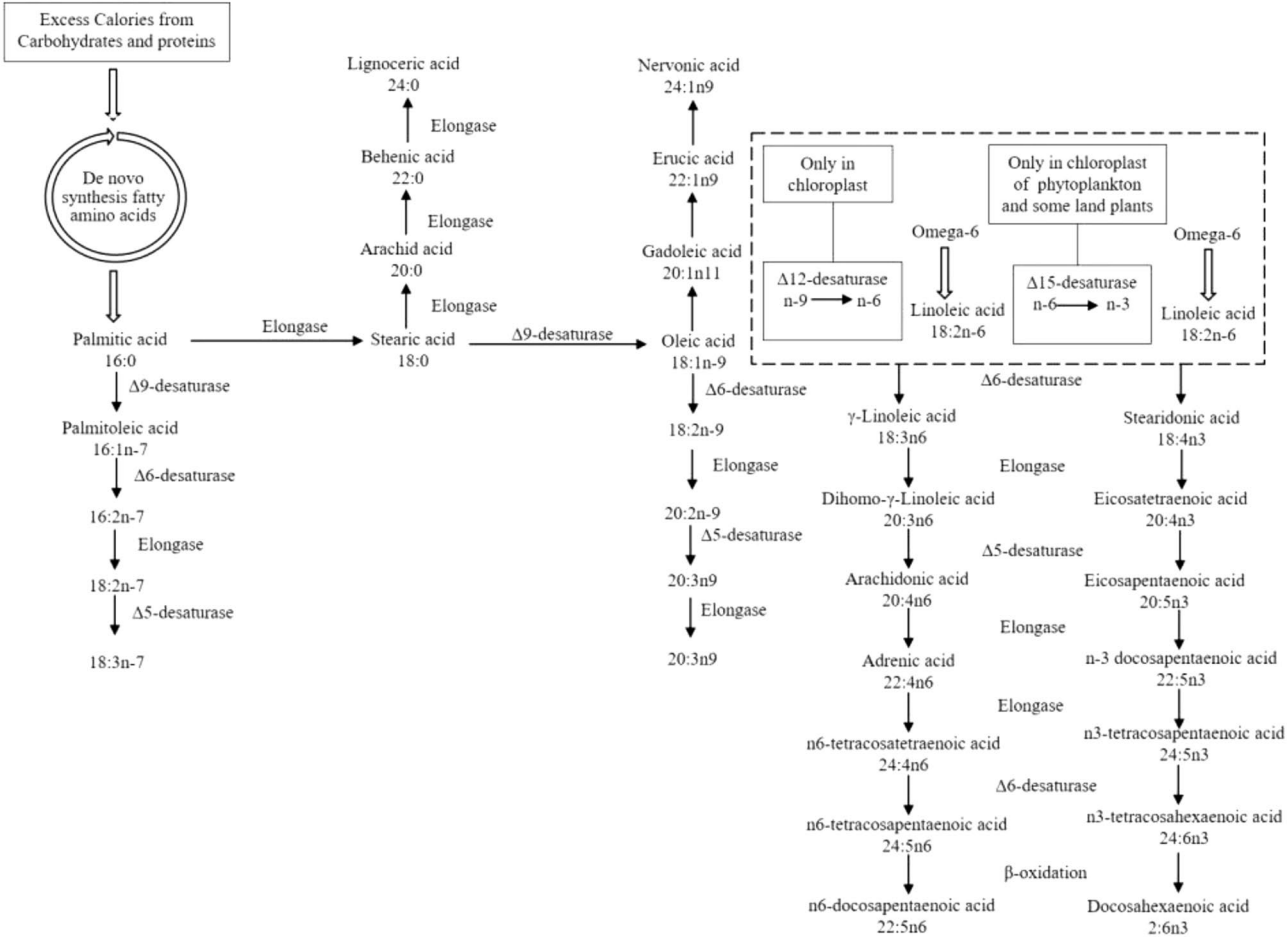
Omega (3 and 6) fatty acid biosynthesis pathways [Adapted from^60,61^ with modifications]. This figure is created by Microsoft PowerPoint software (Version 2016; Microsoft Corporation. Retrieved from https://www.microsoft.com/en-us/microsoft-365/powerpoint).

In general, the main fatty acid components in licorice were 9-Octadecenoic acid (8.72–71.27%), 9,12-Octadecadienoic acid (0.1–56.43%), Hexadecanoic acid (12.84–30.59%), Octadecanoic acid (6.9–15.3%), and Docosanoic acid (0.57–2.77%), Eicosanoic acid (1.07–2.64%), 7-Hexadecenoic acid (0.26–2.62%), and 9,12,15-Octadecatrienoic acid (0.1–2.56%), respectively (Table 1). Using exogenous Si and mycorrhizal inoculation caused changes in the fatty acids (FAs), concerning quantity and quality. Maximum saturated fatty acids (SFA) were observed in response to W_20_Si_1_F_1_ (52.98%). The maximum increase in unsaturated fatty acids (UFA) was observed in W_100_Si_0_F_0_ (76.06%) and W_20_Si_0_F_1_ (75.24%). Polyunsaturated fatty acids (PUFA) and omega 6 showed their maximum quantities, 58.43 and 56.43%, respectively, in response to W_40_Si_0_F_0_. Monounsaturated fatty acids (MUFA) showed their highest content (73.17%) in W_100_Si_0_F_0_, whereas omega-9 (71.71%) acquired maximum value by the W_100_Si_0_F_1_. Since a lower omega6/omega3 ratio reflects better quality in most food products, its best ratio in the present study was observed in response to W_100_Si_0_F_1_ (0.25%), W_20_Si_1_F_1_ (1.88%), and W_100_Si_1_F_0_ (2.46%), respectively (Table 1). The integrated biosynthetic pathways of sugars, fatty acids, as well as omega 3, 6, 7, and 9 are presented in Figs. 4 and 5.

**Table 1:** Fatty acid profile (percent area) of licorice supplemented with Silicon and mycorrhiza.

Hit Name	lipid number	T1*	T2	T3	T4	T5	T6	T7	T8	T9	T10	T11	T12	T13	T14	T15	T16	T17	T18	T19	T20
Pentanoic acid	C05:0	0.22^b^ ± 0.03	0.16^bc^ ± 0.02	0.2^b^ ± 0.03	0.26^b^ ± 0.06	0.19^b^ ± 0.04	0.21^b^ ± 0.05	0.12^bc^ ± 0.02	0.18^b^ ± 0.02	0.23^b^ ± 0.07	0.24^b^ ± 0.04	0^c^ ± 0	0.23^b^ ± 0.04	0.23^b^ ± 0.06	0.19^b^ ± 0.03	0.23^b^ ± 0.03	0.19^b^ ± 0.06	0.13^bc^ ± 0.05	0.22^b^ ± 0.07	0.21^b^ ± 0.05	0.54^a^ ± 0.1
Hexanoic acid	C06:0	0^b^ ± 0	0^b^ ± 0	0.02^a^ ± 0.001	0^b^ ± 0	0^b^ ± 0	0^b^ ± 0	0.02^a^ ± 0.002	0.02^a^ ± 0.001	0^b^ ± 0	0^b^ ± 0	0.02^a^ ± 0.001	0^b^ ± 0	0.01^a^ ± 0	0.02^a^ ± 0.003	0.01^a^ ± 0.001	0^b^ ± 0	0.02^a^ ± 0.003	0^b^ ± 0	0^b^ ± 0	0^b^ ± 0
Octanoic acid	C08:0	0^b^ ± 0	0.06^ab^ ± 0.01	0.06^ab^ ± 0.01	0.04^ab^ ± 0.008	0.05^ab^ ± 0.01	0.06^ab^ ± 0.01	0.09^a^ ± 0.02	0.07^ab^ ± 0.01	0.07^ab^ ± 0.009	0.04^ab^ ± 0.005	0.11^a^ ± 0.02	0.06^ab^ ± 0.008	0.07^ab^ ± 0.01	0.09^a^ ± 0.01	0.06^ab^ ± 0.007	0.04^ab^ ± 0.006	0.1^a^ ± 0.01	0.04^ab^ ± 0.004	0.05^ab^ ± 0.006	0.06^ab^ ± 0.008
Nonanoic acid	C09:0	0^b^ ± 0	0^b^ ± 0	0^b^ ± 0	0^b^ ± 0	0^b^ ± 0	0^b^ ± 0	0^b^ ± 0	0^b^ ± 0	0^b^ ± 0	0^b^ ± 0	1.44^a^ ± 0.11	0^b^ ± 0	0^b^ ± 0	0^b^ ± 0	0^b^ ± 0	0^b^ ± 0	0^b^ ± 0	0^b^ ± 0	0^b^ ± 0	0^b^ ± 0
Decanoic acid	C10:0	0^b^ ± 0	0.11^a^ ± 0.02	0^b^ ± 0	0^b^ ± 0	00^b^ ± 0	0.09^ab^ ± 0.02	0^b^ ± 0	0.11^a^ ± 0.04	0.13^a^ ± 0.05	0^b^ ± 0	0^b^ ± 0	0^b^ ± 0	0^b^ ± 0	0^b^ ± 0	0^b^ ± 0	0^b^ ± 0	00^b^ ± 0	0^b^ ± 0	0^b^ ± 0	0^b^ ± 0
pentadecanoic acid	C15:0	0^c^ ± 0	0.22^b^ ± 0.05	0.17^b^ ± 0.03	0^c^ ± 0	0^c^ ± 0	0^c^ ± 0	0.96^a^ ± 0.12	0^c^ ± 0	0^c^ ± 0	0^c^ ± 0	0^c^ ± 0	0^c^ ± 0	0.06^c^ ± 0.01	0^c^ ± 0	0.95^a^ ± 0.11	0^c^ ± 0	0^c^ ± 0	0^c^ ± 0	0^c^ ± 0	0^c^ ± 0
Hexadecanoic acid	C16:0	14.30^bc^ ± 1.11	17.56^b^ ± 1.49	16.87^b^ ± 1.93	15.26^bc^ ± 2.13	15.6^bc^ ± 1.67	16.55^b^ ± 2.01	15.96^b^ ± 1.55	17.53^b^ ± 1.18	14.89^bc^ ± 1.29	17.51^b^ ± 1.36	18.04^b^ ± 1.75	18.58^b^ ± 1.44	15.69^bc^ ± 1.91	18.64^b^ ± 1.27	16.62^b^ ± 1.33	17.43^b^ ± 1.59	18.7^b^ ± 1.45	17.77^b^ ± 1.68	12.84^c^ ± 1.32	30.59^a^ ± 1.78
7-Hexadecenoic acid	C16:1n9	1.29^b^ ± 0.09	0.38^c^ ± 0.04	0.32^c^ ± 0.02	0.26^c^ ± 0.05	0.39^c^ ± 0.05	0.38^c^ ± 0.03	0.55^bc^ ± 0.07	0.35^c^ ± 0.04	0.75^bc^ ± 0.06	0.37^c^ ± 0.03	0.84^bc^ ± 0.08	1.22^b^ ± 0.14	2.59^a^ ± 0.22	1.41^b^ ± 0.13	0.35^c^ ± 0.05	0.73^bc^ ± 0.06	0.49^bc^ ± 0.08	1.44^b^ ± 0.11	0.53^bc^ ± 0.09	2.62^a^ ± 0.21
Heptadecanoic acid	C17:0	0.13^b^ ± 0.03	0.18^b^ ± 0.05	0.16^b^ ± 0.07	0.14^b^ ± 0.02	0.18^b^ ± 0.05	0.16^b^ ± 0.03	0.22^b^ ± 0.06	0.17^b^ ± 0.07	0.17^b^ ± 0.04	0.18^b^ ± 0.05	0.38^b^ ± 0.06	0.17^b^ ± 0.02	0.22^b^ ± 0.04	0.22^b^ ± 0.03	0.15^b^ ± 0.04	0.15^b^ ± 0.02	0.19^b^ ± 0.07	1.14^a^ ± 0.12	0^c^ ± 0	0.34^b^ ± 0.08
Octadecanoic acid	C18:0	6.99^c^ ± 0.15	7.85^c^ ± 0.18	7.73^c^ ± 0.21	7.94^c^ ± 0.24	10.19^b^ ± 0.31	10.21^b^ ± 0.19	8.25^c^ ± 0.28	9.58^c^ ± 0.33	7.78^c^ ± 0.17	10.34^b^ ± 0.41	9.04^c^ ± 0.25	10.96^b^ ± 0.36	9.48^c^ ± 0.22	10.77^b^ ± 0.35	12.23^b^ ± 0.26	12.15^b^ ± 0.24	9.94^b^ ± 0.19	10.69^b^ ± 0.28	6.9^c^ ± 0.13	15.3^a^ ± 0.33
9-Octadecenoic acid	C18:1n9	71.27^a^ ± 3.66	67.57^a^ ± 2.59	70.64^a^ ± 3.97	69.67^a^ ± 3.53	23^d^ ± 1.86	22.85^d^ ± 1.98	22.68^d^ ± 2.61	23.51^d^ ± 3.11	24.64^d^ ± 2.54	23.08^d^ ± 2.88	17.77^d^ ± 2.31	20.77^d^ ± 2.12	8.72^e^ ± 1.11	21.27^d^ ± 1.94	23.84^d^ ± 2.37	19.28^d^ ± 1.88	60.5^b^ ± 4.15	19.67^d^ ± 2.44	35.68^c^ ± 2.91	41.05^c^ ± 3.24
9,12-Octadecadienoic acid	C18:2n6	2.79^c^ ± 0.08	1.12^c^ ± 0.05	0.1^c^ ± 0.02	2.09^c^ ± 0.33	43.53^b^ ± 3.04	45.76^b^ ± 2.69	46.33^b^ ± 3.05	44.68^b^ ± 2.78	45.44^b^ ± 3.66	43.2^b^ ± 3.71	45.08^b^ ± 2.53	40.43^b^ ± 1.94	56.43^a^ ± 3.33	41.06^b^ ± 2.94	40.4^b^ ± 3.01	45.56^b^ ± 2.58	6.06^c^ ± 0.13	42.81^b^ ± 2.14	37.38^b^ ± 1.66	1.98^c^ ± 0.25
9,12,15-Octadecatrienoic acid	C18:3n3	0.1^bc^ ± 0.03	0.45^b^ ± 0.08	0.39^b^ ± 0.06	0.34^b^ ± 0.08	2.33^a^ ± 0.15	0.1^bc^ ± 0.03	0.35^b^ ± 0.08	0.27^b^ ± 0.08	0.47^b^ ± 0.12	1.17^ab^ ± 0.09	0.12^bc^ ± 0.05	2.34^a^ ± 0.11	2^a^ ± 0.14	1.51^ab^ ± 0.12	1.92^a^ ± 0.13	1.47^ab^ ± 0.13	0.7^b^ ± 0.06	2.56^a^ ± 0.12	1.66^ab^ ± 0.11	1.06^ab^ ± 0.08
Eicosanoic acid	C20:0	1.07^c^ ± 0.08	1.37^b^ ± 0.11	1.21^c^ ± 0.09	1.14^c^ ± 0.08	1.42^b^ ± 0.12	1.43^b^ ± 0.11	1.49^b^ ± 0.12	1.28^bc^ ± 0.07	2.4^a^ ± 0.08	1.15^c^ ± 0.07	2.33^a^ ± 0.13	1.3^b^ ± 0.09	1.65^b^ ± 0.14	2.45^a^ ± 0.12	1.37^b^ ± 0.11	1.33^b^ ± 0.11	1.31^b^ ± 0.14	1.1^c^ ± 0.08	1.46^b^ ± 0.15	2.64^a^ ± 0.13
11-Eicosenoic acid	C20:1n9	0.62^a^ ± 0.09	0^b^ ± 0	0.05^b^ ± 0.02	0.66^a^ ± 0.07	0^b^ ± 0	0^b^ ± 0	0^b^ ± 0	0^b^ ± 0	0^b^ ± 0	0^b^ ± 0	0^b^ ± 0	0^b^ ± 0	0^b^ ± 0	0^b^ ± 0	0^b^ ± 0	0^b^ ± 0	0^b^ ± 00	0^b^ ± 0	0^b^ ± 0	0^b^ ± 0
6,11-Eicosadienoic acid	C20:2n9	0^b^ ± 0	0^b^ ± 0	0^b^ ± 0	0^b^ ± 0	0^b^ ± 0	0^b^ ± 0	0^b^ ± 0	0^b^ ± 0	0^b^ ± 0	0^b^ ± 0	0^b^ ± 0	0^b^ ± 0	0^b^ ± 0	0.21^a^ ± 0.05	0^b^ ± 0	0^b^ ± 0	0^b^ ± 0	0^b^ ± 0	0^b^ ± 0	0^b^ ± 0
Docosanoic acid	C22:0	0.57^c^ ± 0.12	1.1^b^ ± 0.09	0.64^c^ ± 0.11	1.46^b^ ± 0.12	1.58^b^ ± 0.13	1.34^b^ ± 0.08	1.28^b^ ± 0.14	1.53^b^ ± 0.13	1.23^b^ ± 0.11	1.7^b^ ± 0.12	2.36^a^ ± 0.11	2.77^a^ ± 0.14	1.43^b^ ± 0.15	1.41^b^ ± 0.13	0.7^c^ ± 0.04	0.66^c^ ± 0.08	0.84^c^ ± 0.09	0.72^c^ ± 0.06	0.84^c^ ± 0.07	1.67^b^ ± 1.11
Tricosanoic acid	C23:0	0.06^c^ ± 0.01	0.63^b^ ± 0.11	0.07^c^ ± 0.02	0.06^c^ ± 0.01	0.08^c^ ± 0.02	0.06^c^ ± 0.02	0.06^c^ ± 0.02	0.06^c^ ± 0.01	0.07^c^ ± 0.02	0.05^c^ ± 0.01	0.25^b^ ± 0.07	0.06^c^ ± 0.01	0.06^c^ ± 0.02	0.07^c^ ± 0.02	0.06^c^ ± 0.01	0.07^c^ ± 0.01	0.07^c^ ± 0.01	0.08^c^ ± 0.02	1.67^a^ ± 0.08	0^c^ ± 0
Tetracosanoic acid	C24:0	0.41^bc^ ± 0.05	0.42^bc^ ± 0.04	0.37^c^ ± 0.07	0.38^c^ ± 0.06	0.59^b^ ± 0.08	0.38^c^ ± 0.05	0.49^b^ ± 0.07	0.35^c^ ± 0.03	0.4^bc^ ± 0.06	0.5^b^ ± 0.05	1.14^b^ ± 0.09	0.4^bc^ ± 0.14	0.44^b^ ± 0.11	0.42^bc^ ± 0.13	0.47^b^ ± 0.11	0.56^b^ ± 0.12	0.47^b^ ± 0.11	0.84^b^ ± 0.15	0.64^b^ ± 0.13	1.83^a^ ± 0.22
15-Tetracosenoic acid	C24:1n9	0^b^ ± 0	0^b^ ± 0	0.7^a^ ± 0.08	0^b^ ± 0	0^b^ ± 0	0^b^ ± 0	0^b^ ± 0	0^b^ ± 0	0.84^a^ ± 0.17	0^b^ ± 0	0^b^ ± 0	0^b^ ± 0	0^b^ ± 0	0^b^ ± 0	0^b^ ± 0	0^b^ ± 0	0^b^ ± 0	0^b^ ± 0	0^b^ ± 0	0^b^ ± 0
Pentadecanoic acid	C25:0	0.13^b^ ± 0.06	0.07^b^ ± 0.02	0.05^bc^ ± 0.01	0.1^b^ ± 0.03	0.08^b^ ± 0.02	0.04^bc^ ± 0.01	0.18^b^ ± 0.05	0.08^b^ ± 0.02	0.07^b^ ± 0.02	0.04^bc^ ± 0.01	0.35^a^ ± 0.12	0^c^ ± 0	0.05^bc^ ± 0.01	0.07^b^ ± 0.01	0^c^ ± 0	0.06^b^ ± 0.01	0^c^ ± 0	0^c^ ± 0	0^c^ ± 0	0^c^ ± 0
Hexacosanoic acid	C26:0	0^c^ ± 0	0.09^b^ ± 0.02	0.05^c^ ± 0.01	0.04^c^ ± 0.01	0.11^b^ ± 0.04	0^c^ ± 0	0.12^b^ ± 0.05	0^c^ ± 0	0^c^ ± 0	0^c^ ± 0	0.38^a^ ± 0.08	0^c^ ± 0	0^c^ ± 0	0^c^ ± 0	0^c^ ± 0	0^c^ ± 0	0^c^ ± 0	0^c^ ± 0	0^c^ ± 0	0^c^ ± 0
Total fatty acid (TFA)	TFA	99.73^a^ ± 4.31	99.33^ab^ ± 2.55	99.79^a^ ± 3.67	99.84^a^ ± 3.82	99.3^ab^ ± 3.26	99.61^a^ ± 3.51	99.14^ab^ ± 3.44	99.78^a^ ± 3.19	99.56^a^ ± 3.26	99.58^a^ ± 4.24	99.64^a^ ± 2.73	99.29^ab^ ± 3.56	99.14^ab^ ± 3.14	99.79^a^ ± 2.91	99.36^ab^ ± 3.21	99.66^a^ ± 4.11	99.51^a^ ± 3.55	99.1^ab^ ± 3.64	99.86^a^ ± 3.71	99.68^a^ ± 4.33
Saturated fatty acid (SFA)	SFA	23.66^b^ ± 1.12	29.81^b^ ± 1.33	27.59^b^ ± 1.42	26.82^b^ ± 1.28	30.06^b^ ± 2.08	30.52^b^ ± 1.09	29.23^b^ ± 2.03	30.97^b^ ± 2.44	27.42^b^ ± 2.01	31.75^b^ ± 2.17	35.83^b^ ± 3.02	34.52^b^ ± 2.44	29.39^b^ ± 2.11	34.33^b^ ± 2.88	32.86^b^ ± 2.05	32.63^b^ ± 1.79	31.77^b^ ± 1.98	32.61^b^ ± 1.67	24.62^b^ ± 2.16	52.98^a^ ± 3.33
Unsaturated fatty acid (USFA)	USFA	76.06^a^ ± 4.66	69.51^b^ ± 5.04	72.2^ab^ ± 3.65	73.02^ab^ ± 4.11	69.24^b^ ± 2.77	69.09^b^ ± 3.82	69.91^b^ ± 3.84	68.81^b^ ± 2.95	72.14^ab^ ± 4.06	67.83^b^ ± 3.27	63.81^bc^ ± 3.56	64.77^bc^ ± 4.08	69.75^b^ ± 4.03	65.45^bc^ ± 5.01	66.5^b^ ± 4.55	67.03^b^ ± 2.98	67.74^b^ ± 3.78	66.49^b^ ± 4.05	75.24^a^ ± 4.11	46.7^c^ ± 3.32
Monounsaturated fatty acid (MUFA)	MUFA	73.17^a^ ± 4.77	67.95^a^ ± 4.52	71.71^a^ ± 3.99	70.59^a^ ± 3.68	23.38^c^ ± 1.25	23.22^c^ ± 1.34	23.24^c^ ± 2.13	23.86^c^ ± 1.22	26.23^c^ ± 2.55	23.45^c^ ± 1.24	18.61^de^ ± 1.33	22^ cd^ ± 1.77	11.31^e^ ± 1.21	22.68^c^ ± 1.11	24.19^c^ ± 2.11	20.01^d^ ± 2.88	60.98^ab^ ± 5.12	21.12^d^ ± 2.14	36.2^bc^ ± 3.44	43.67^b^ ± 2.08
Polyunsaturated fatty acid (PUFA)	PUFA	2.89^d^ ± 1.12	1.57^e^ ± 0.13	0.49^e^ ± 0.15	2.43^d^ ± 0.21	45.86^b^ ± 3.64	45.86^b^ ± 3.15	46.68^b^ ± 2.61	44.95^b^ ± 3.61	45.91^b^ ± 4.01	44.38^b^ ± 2.67	45.2^b^ ± 3.26	42.77^b^ ± 2.98	58.43^a^ ± 3.66	42.78^b^ ± 2.16	42.31^b^ ± 2.68	47.02^b^ ± 2.92	6.76^d^ ± 3.41	45.38^b^ ± 3.63	39.04^c^ ± 4.09	3.04^d^ ± 0.25
omega-3	omega-3	0.1^c^ ± 0.03	0.45^bc^ ± 0.06	0.39^bc^ ± 0.06	0.34^bc^ ± 0.08	2.33^a^ ± 0.11	0.1^c^ ± 0.03	0.35^bc^ ± 0.07	0.27^bc^ ± 0.06	0.47^bc^ ± 0.08	1.17^b^ ± 0.09	0.12^c^ ± 0.05	2.34^a^ ± 0.59	2^a^ ± 0.32	1.51^ab^ ± 0.18	1.92^a^ ± 0.24	1.47^ab^ ± 0.32	0.7^b^ ± 0.13	2.56^a^ ± 0.44	1.66^ab^ ± 0.41	1.06^b^ ± 0.09
omega-6	omega-6	2.79^ cd^ ± 0.13	1.12^d^ ± 0.14	0.1^d^ ± 0.05	2.09^ cd^ ± 0.09	43.53^ab^ ± 2.33	45.76^a^ ± 3.28	46.33^a^ ± 3.71	44.68^ab^ ± 3.55	45.44^a^ ± 3.91	43.2^ab^ ± 3.11	45.08^ab^ ± 2.69	40.43^b^ ± 3.46	56.43^a^ ± 4.58	41.06^b^ ± 3.42	40.4^b^ ± 2.55	45.56^a^ ± 2.63	6.06^c^ ± 0.21	42.81^ab^ ± 4.52	37.38^b^ ± 4.08	1.98^ cd^ ± 0.14
omega-9	omega-9	73.17^a^ ± 5.44	67.95^ab^ ± 5.26	71.71^a^ ± 5.22	70.59^a^ ± 4.55	23.38^d^ ± 1.81	23.22^d^ ± 1.29	23.24^d^ ± 1.16	23.86^d^ ± 1.19	26.23^d^ ± 2.15	23.45^d^ ± 1.69	18.61^de^ ± 2.14	22^de^ ± 2.03	11.31^e^ ± 1.17	22.89^d^ ± 2.33	24.19^d^ ± 1.56	20.01^de^ ± 1.99	60.98^b^ ± 3.65	21.12^de^ ± 2.13	36.2^ cd^ ± 3.66	43.67^c^ ± 3.67
omega-6/omega-3	n6/n3	27.94^e^ ± 2.09	2.46f. ± 0.33	0.25f. ± 0.06	6.2f. ± 0.12	18.68^ef^ ± 3.13	457.61^a^ ± 7.66	132.37^ cd^ ± 6.51	165.49^c^ ± 6.15	97.52^d^ ± 4.87	36.8^e^ ± 4.66	381.73^b^ ± 7.28	17.28^ef^ ± 1.69	28.22^e^ ± 2.33	27.17^e^ ± 2.35	21.07^ef^ ± 2.18	31.01^e^ ± 2.54	8.67f. ± 1.14	16.72^ef^ ± 1.85	22.47^ef^ ± 2.05	1.88f. ± 0.34

The compounds of fatty acids were identified by software of the GC–MS apparatus with a comparison of retention times and fragmentation patterns of the related peaks with those reported in the libraries of Wily and NIST. According to the analysis of variance that triple effects of fungi, drought levels and Si showed significant difference, slice method used for mean comparisons. Mean values with the same letters within a row are not significantly different (*p* < 0.05), Tukey test.

*Treatments (T) are shown with numbers; 1: W_100_ Si_0_ F_0_; 2: W_100_ Si_1_ F_0_; 3: W_100_ Si_0_ F_1_; 4: W_100_ Si_1_ F_1_; 5: W_80_ Si_0_ F_0_; 6: W_80_ Si_1_ F_0_; 7: W_80_ Si_0_ F_1_; 8: W_80_ Si_1_ F_1_; 9: W_60_ Si_0_ F_0_; 10: W_60_ Si_1_ F_0_; 11: W_60_ Si_0_ F_1_; 12: W_60_ Si_1_ F_1_; 13: W_40_ Si_0_ F_0_; 14: W_40_ Si_1_ F_0_; 15: W_40_ Si_0_ F_1_; 16: W_40_ Si_1_ F_1_;17: W_20_ Si_0_ F_0_; 18: W_20_ Si_1_ F_0_; 19: W_20_ Si_0_ F_1_; 20: W_20_ Si_1_ F_1_.

### Principle component analysis of sugars and fatty acids of *G. glabra* under a combination of mycorrhiza, silicon, and drought levels

**Figure 6:**
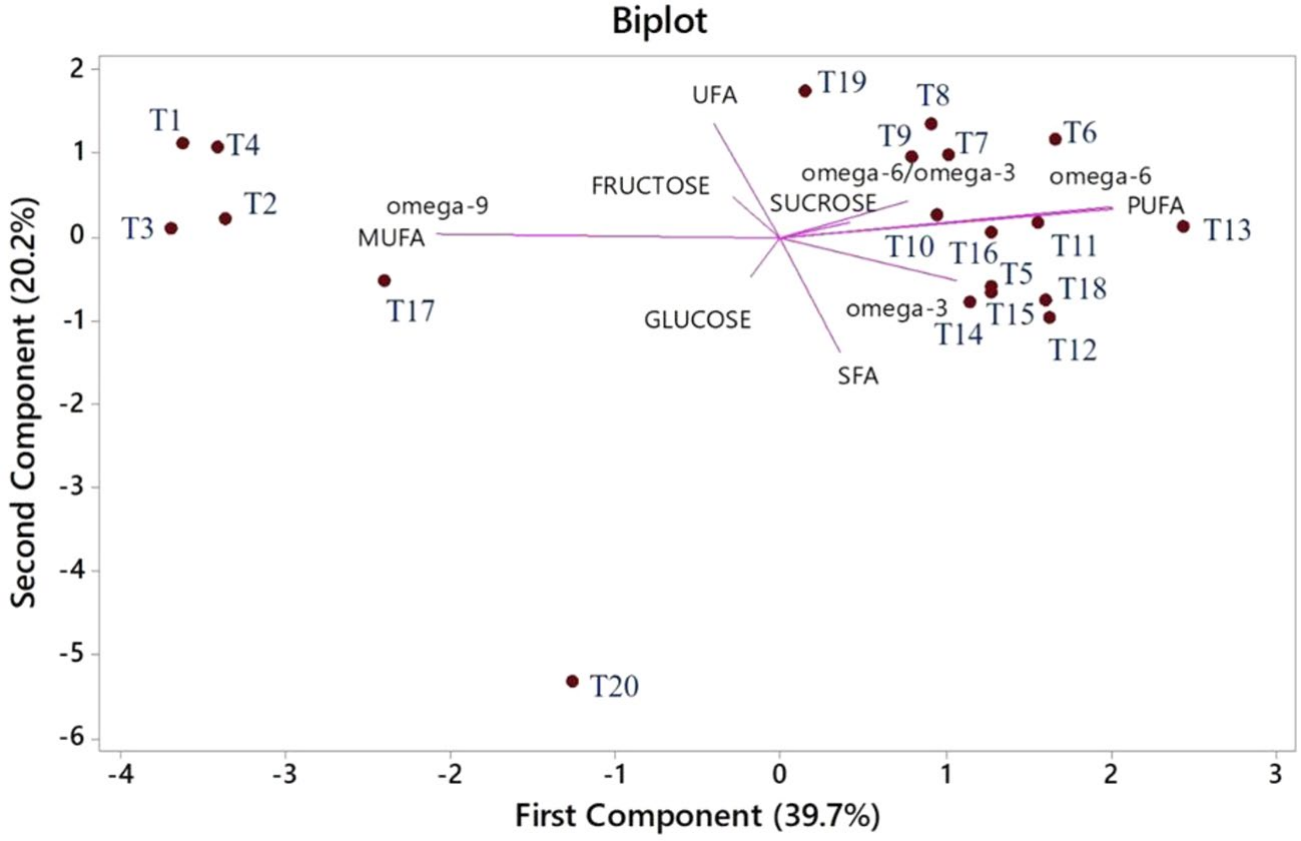
Principal component analysis of studied treatments on sugars and fatty acids of licorice. *SFA* saturated fatty acid, *USFA* unsaturated fatty acid, *MUFA* monounsaturated fatty acid, *PUFA* polyunsaturated fatty acid. *Treatments (T) are shown with numbers; 1: W_100_ Si_0_ F_0_; 2: W_100_ Si_1_ F_0_; 3: W_100_ Si_0_ F_1_; 4: W_100_ Si_1_ F_1_; 5: W_80_ Si_0_ F_0_; 6: W_80_ Si_1_ F_0_; 7: W_80_ Si_0_ F_1_; 8: W_80_ Si_1_ F_1_; 9: W_60_ Si_0_ F_0_; 10: W_60_ Si_1_ F_0_; 11: W_60_ Si_0_ F_1_; 12: W_60_ Si_1_ F_1_; 13: W_40_ Si_0_ F_0_; 14: W_40_ Si_1_ F_0_; 15: W_40_ Si_0_ F_1_; 16: W_40_ Si_1_ F_1_;17: W_20_ Si_0_ F_0_; 18: W_20_ Si_1_ F_0_; 19: W_20_ Si_0_ F_1_; 20: W_20_ Si_1_ F_1_.

In the biplot of PC analysis, the first two PCs explained 59.9% of variations in glucose, sucrose, fructose, and fatty acid contents as a result of the treatments (Fig. 6). The first PC explained 39.7% of the variation as it comprised sucrose, omega-6/omega-3, omega-6, PUFA, omega-3, and SFA. In contrast, the second PC accounted for 20.2% of the variations, comprising fructose, glucose, UFA, MUFA, and omega-9. In the present study, the projection of vectors, representing treatments, on the two detected PCs in the bi-plot, divided the treatments into two distinct groups. The first group comprised W_80_Si_0_F_0_, W_80_Si_1_F_0_, W_60_Si_0_F_0_, W_60_Si_1_F_1_, W_40_Si_0_F_0_, W_40_Si_1_F_0_, W_40_Si_0_F_1_, W_20_Si_1_F_0_, W_80_Si_0_F_1_, W_80_Si_1_F_1_, W_60_Si_1_F_0_, W_60_Si_0_F_1_, W_40_Si_1_F_1_, and W_20_Si_0_F_1_ and in association with the first PC, it linked with soluble carbohydrate contents. Meanwhile, the second group comprised W_100_Si_0_F_0_, W_100_Si_0_F_1_, W_100_Si_1_F_1_, W_100_Si_1_F_0_, W_20_Si_0_F_0_, and W_20_Si_1_F_1_ because these treatments had a greater association with the second PC-linked traits. The results indicated that each treatment group had a better performance when scattered between the PC vectors (Fig. 6).

## Discussion

### Carbohydrates

Sugars are one of the most important quality parameters in licorice root. Present results indicated that sugar storage in licorice roots can be induced by AM symbiosis, which is a good strategy to overcome drought conditions. In addition to increasing product quality, sugars can protect membrane integrity, inhibit structural changes to insoluble proteins and maintain osmotic equilibrium in plant cells under harsh environments^27^. In a study on *Triticum aestivum*, *Rhizophagus intraradices* was used as an AMF to assess how it affected sugar metabolism. It was observed that sugar and starch contents increased in mycorrhizal wheat plants. Their results revealed changes in sugar metabolism through the modulation of starch phosphorylase, sucrose synthase, and sucrose-phosphate synthase. Variations in the sugar contents of non-mycorrhizal inoculated plants and mycorrhizal inoculated plants were ultimately reflected in the accumulation of reducing sugars that can scavenge ROS, which is critical under stressed conditions. Thus, inoculated plants could better tolerate drought stress^28^. Sucrose, as an osmolyte, is mainly produced in the leaves and is the primary form of carbohydrate. Mycorrhizal inoculated licorice in the current study, showed a higher sucrose concentration, that are aimed for long-distance transportation in the process of supplying the enormous demand for sugars under water-deficit stress^29^.

Similar to the present study, higher sucrose content in inoculated plants was also observed in *Pinus tabulaeformis*^30^ and *Sorghum bicolor*^31^. Osmolytes can maintain the integrity of membranes against the negative impacts of drought stress. Sinks usually trigger a demand for sugars in fungi to obtain energy from shoot tissues, which is followed by the hydrolysis of starch to sugars in seedlings inoculated with mycorrhizal fungi. These can assist in upholding osmotic equilibrium in plant cells, and thus preserve membrane integrity, whereby mycorrhizal fungi can increase the sugar content of the host plant^32^. Mycorrhizal colonization has often resulted in the accumulation of osmotic solutes under drought stress by modifying the osmotic balance through the carbohydrate profile, AMF can optimize physiological processes in the host plant^33^. In clover, regardless of the soil moisture, the leaves of AMF seedlings, as inoculated by *P. occultum*, had considerably greater levels of fructose, glucose, and sucrose than those inoculated with *F. mosseae*. AMF colonization by *F. mosseae* and *P. occultum* caused a significant increase in sucrose, fructose, and glucose contents in plant leaf, despite drought stress, meaning that osmolytes protected and stabilized plant macromolecules, thereby improving the ability of plants in resisting drought tolerance by osmotic adjustment^34^. In addition to the effect of AMF on the carbohydrates content, mineral nutrition such as Si, as evaluated in the current study, could be also effective on plant metabolism.

A study on sugarcane showed that Si plays a role in the synthesis and storage of sucrose. Low Si quantities can combine effectively and physically with sucrose, thereby preventing invertase from binding to its substrate. Even when sucrose is inverted, the putative fructose-silicate structure remains in its original state, mostly by prohibiting bacteria from metabolizing fructose. Since Si was used to deal with drought stress, Si supplementation might contribute to a higher water-uptake capacity by plants^35^. Higher Si levels led to greater soluble sugar concentrations. In Si-treated plants, however, a higher rate of photosynthesis occurred probably due to a simultaneous rise in soluble sugars and starch concentrations. Since epidermal cell walls are filled with a solid layer of Si, they are inherently strong barriers against fungal infections and water loss^36^. Also, since the drought stress causes minerals’ insufficiency, deficiencies in plant nutrients have been reportedly alleviated by Si treatments. Another study showed that Si reduced the sucrose content in barley leaves under drought stress, while it enhanced the sucrose level in barley grains^37^. In *Oryza sativa*, sucrose, fructose, and glucose contents decreased in Si-treated seedlings. Si may restrict sugar transportation in the phloem of roots and, thus, reduce photosynthesis in the shoots, as evidenced by their low sucrose content^38^.

In addition to sugars, which are known as basic osmolytes, the main type of carbohydrate storage in plants is starch. Carbohydrates are necessary for osmoprotection and carbon storage in plants as soon as they are exposed to drought stress. To meet this necessity, there is usually an increase in starch and sucrose catabolism, as well as sucrose metabolism in enzymatic activity, resulting in changes to carbohydrate metabolism that usually concerns glucose, sucrose, dextrins, and maltose production. Starch can be reduced to dextrins and maltose, respectively, via enzymes α- and β-amylases. Meanwhile, extra carbon can be stored in two ways, either as soluble sugars in vacuoles, as polymeric forms such as starch in plastids, or as oil molecules in vesicles^39^. Starch synthase, branching, and debranching enzymes are required for starch synthesis, while β- amylase, and α-amylase are precursors of starch metabolism. The abiotic stress response in plants is influenced by starch metabolism, although starch degradation has reportedly decreased because of this in some crops^40^. Enzymatic degradation of plant polymeric carbohydrates like starch and cellulose into simple sugars can sometimes lead to the provision of valuable end-products in the industry^41^. Similar to the present results, where a lower level of irrigation led to lower starch content, Si-treated barley plants reportedly varied distinctively in their quantity of starch, which enhanced dramatically under drought stress. It was observed that applying Si to barley, with the addition of osmotic stress-induced Si transporters, causes Si to be transported to the shoots, thereby having reason to increase starch content and to regulate ABA homeostasis, with the ultimate effect of improving plant tolerance to stress^42^. In barley, drought stress was seen to have smaller effects on sugars, particularly insoluble starch, important tricarboxylic acid cycle metabolites, 2-oxoglutarate, and fumarate, as well as glycolytic intermediates of glucose-6phosphate, fructose-6phosphate, and 3-Phosphoglyceric acid. Drought stress caused carbohydrates to accumulate in the leaves and be a replacement for osmotic molecules. Drought stress is known to affect glucose metabolism and starch availability. Under drought stress, starch globules tend to aggregate and metabolic rearrangements usually occur^43^.

### Fatty acids

In organisms, FAs are are the major components of membrane lipids, while variations in FAs saturation levels and compositions are linked to plant tolerance against drought^44,45^. Plants can aim at compensating for water loss in soils with low water levels by stomatal closure, which inhibits CO_2_ availability for photosynthesis. Variations in fatty acid content could result from their capacity to be used as a carbon source for fatty acid production. Fatty acid composition can vary under drought stress situations^46^. In a study on *Folsomia candida*, drought-induced fatty acid desaturation, together with membrane-protecting cryoprotective accumulation, were designated as key forms of physiological adaptations to tolerance against desiccation. Acclimation to drought has reportedly resulted in changes to membrane fatty acids, along with a considerable decrease in cell membrane transition temperature as this can occur expectedly in the process of plant adaption to drought^47^. Researchers discovered lower levels of linoleic acid in several *Brassica* species when they were subjected to drought stress^48^. Drought stress tends to elevate ROS levels, and plants utilize a variety of ways to cope with the adverse outcomes of drought. Some plants can change their oil content and compositions, to compensate for variations in water relations within the cells and organs. Drought stress can cause an increase in SFAs and a decrease in UFAs, which reduces the fluidity of cell membrane lipids. A high concentration of UFAs should be viewed as a key mechanism for improving plant tolerance against drought^49^.

Since drought stress can affect the fatty acid synthesis pathway, it also has a considerable impact on Delta12-fatty-acid desaturase, a key enzyme in fatty acid synthesis^50^.

Minerals are also effective in mitigating water-deficit stress by affecting fatty acids. A previous study showed how Si has notable impacts on oil quantity, SFA, and the unsaturated fatty acid profile of flax^51^. In line with the present study, drought stress affected rapeseed cultivars differently^52^. A study on *Hordeum vulgare* showed that the application of Si on stressed plants enhanced the ratio of UFA/SFA in drought-tolerant cultivars, compared to non-Si-amended treatments^53^. Another study showed that the composition of fatty acids can alter membrane fluidity and activities which is important under stressed conditions. Adding Si to drought treatments significantly reduced stearic and oleic acid levels. A study on *Brassica napus* revealed how drought stress reduced linolenic acid (%) but increased oleic acid (%)^54,55^. Another strategy for mitigating drough stress is AMF application that is effective in nutrient availability for plants^56^. Research on *Poncirus trifoliata* showed that AMF inoculation caused a substantial increase in methyl oleate, methyl linoleate, and methyl linolenate concentrations in the roots, despite severe drought conditions. In contrast, methyl stearate levels decreased in the roots under severe drought conditions. These variations in the profile of FAs in mycorrhized roots have led to a higher unsaturation index that reportedly reduced oxidative damage^57^. This causes better resistance under harsh stressed conditions. In the genome of *Rhizophagus irregularis*, researchers have discovered genes that encode enzymes for the breakdown and elongation of FAs. Furthermore, FAs in host plants are delivered to AMF for the maintenance of mycorrhizal colonization, which is mediated by the adenosine triphosphate-binding cassette transporter. Thus, FAs are crucial for AM development and for triggering plant resistance to abiotic stress. AMF could influence the composition of FAs and the quantity of UFAs to improve drought resistance by host plants which is similary observed in the current results^58^.

## Conclusion

A major challenge that licorice production currently faces in the industry is drought stress that causes undesirable variations in various metabolites’ contents and compositions. The protective effects of Si and AMF treatments appear to be connected with the accumulation of primary and secondary metabolites and mineral absorption, thereby improving plant quality so that licorice production could remain partly unaffected despite water-deficit situations. As an unfavorable metabolite profile tends to reduce the quality of licorice roots and, thus, makes it almost unsuitable for licorice processing industries, so the provision of appropriate and adequate mineral nutrition, such as exogenous Si and biofertilizers such as arbuscular mycorrhiza, could offer a suitable approach to reduce the adverse effects of water-deficits where licorice is cultivated.. These alterations to carbohydrate and fatty acid profile and contents were to better protection of the plant against drought stress. The current findings provide a practical foundation for the use of Si fertilizers and AMF to better enable licorice production where irrigation systems lean toward a policy of water conservation. Exogenous application of AMF and Si can have synergistic roles in mitigating the adverse effects of water-deficit by improving quantity and quality of sugars and the omega fatty acids in licorice. These findings bring prospective insight into world water deficit crisis conquering.

## Data Availability

The datasets used and/or analysed during the current study are available from the corresponding author on reasonable request.
